# Pathological Grade-Associated Transcriptome Profiling of lncRNAs and mRNAs in Gliomas

**DOI:** 10.3389/fonc.2020.00253

**Published:** 2020-03-10

**Authors:** Junlong Sun, Rui Jiang, Mengruo Song, Junzhong Yao, Shiqiang Hou, Yunhua Zhu, Xiang Ji, Hao Sheng, Zhongyu Tang, Qianqian Liu, Zhongzheng Jia, Wei Shi, Jinlong Shi

**Affiliations:** ^1^Jiangsu Clinical Medicine Center of Tissue Engineering and Nerve Injury Repair and Department of Neurosurgery, The Affiliated Hospital of Nantong University, Nantong, China; ^2^Department of Neurosurgery, Shanghai Jiao Tong University School of Medicine Affiliated Renji Hospital, Shanghai, China; ^3^Department of Clinic Research Center, The Affiliated Hospital of Nantong University, Nantong, China; ^4^Department of Neurosurgery, Chuzhou Clinical College of Anhui Medical University, The First Peoples Hospital Chuzhou, Chuzhou, China; ^5^Medical Image Center, The Affiliated Hospital of Nantong University, Nantong, China

**Keywords:** glioma, mRNA, lncRNA, high-throughput sequencing, transcriptome

## Abstract

The aim of the present study was to explore the expression profiles of lncRNAs and mRNAs in glioma patients and to elucidate any potential relationship between lncRNAs and mRNAs in glioma. High-throughput transcriptome sequencing of mRNAs and lncRNAs from six normal tissues and 16 glioma tissues (grade II, six cases; grade III, four cases; and grade IV, six cases) was performed. Series test of cluster (STC) analysis was used to screen significant trending models associated with glioma. Gene co-expression networks were constructed for the differentially expressed lncRNAs and mRNAs, and gene-ontology (GO) and pathway-enrichment analyses were further performed. Quantitative real-time PCR was performed to validate the five most differentially expressed lncRNAs and mRNAs. After filtering the raw sequencing data, we found 578 lncRNAs and 3,216 mRNAs that were significantly dysregulated in glioma (fold change ≥ 2, *p* < 0.05). Twenty model profiles of lncRNA and 10 model profiles of mRNA were summarized, and three patterns of lncRNAs and two patterns of mRNAs were of clinical significance. Three gene co-expression networks between mRNAs and lncRNAs were built to clarify the relationship between lncRNAs and mRNAs in glioma. GO and pathway analyses indicated that the differentially expressed lncRNAs and mRNAs were enriched in several biological processes and signaling pathways associated with tumorigenesis. Both lncRNAs and mRNAs exhibited dynamic differential expression profiles that indicated their potential roles in different degrees of glioma malignancy. A series of bioinformatics analyses indicated that most of these lncRNAs and mRNAs are involved in important biological processes and pathways associated with the pathogenesis of glioma. These results provide potential directions and valuable resources for future investigations via the comprehensive integration of these lncRNAs and mRNAs.

## Introduction

Gliomas are the most common type of primary brain tumor (1) representing 75% of all malignant primary central-nervous-system (CNS) tumors in adults (2). In the updated 2016 version of the World Health Organization (WHO) classification of CNS tumors, gliomas are divided into circumscribed gliomas (WHO grade I) and diffusely-infiltrating gliomas (whether astrocytic or oligodendroglial, WHO grades II–IV) (3). Compared to circumscribed gliomas, diffusely-infiltrating gliomas exhibit a more relentless malignant progression, a reduced efficacy to various therapeutic approaches, and a higher risk of recurrence (4). Despite efforts to promote various new therapies and advances in the research of tumor biology, the prognosis for patients with gliomas, especially diffusely-infiltrating gliomas, is still bleak (2, 5). This is mainly due to a lack of accurate biomarkers and a poor understanding of the pathogenesis of gliomas, which leads to delayed diagnoses and ineffective therapeutic outcomes. Therefore, there is an urgent need to better understand the mechanisms underlying glioma and to find potential biomarkers and accurate therapeutic targets.

Long non-coding RNAs (lncRNAs) account for a major class of non-coding RNAs (ncRNAs) and measure a length >200 nucleotides (6, 7). Recent studies have demonstrated that lncRNAs may be involved in gene expression via four different processes: epigenetic regulation, translational regulation, transcriptional regulation, and post-transcriptional regulation (8–12). Increasing evidence has suggested that lncRNAs are vital epigenetic regulators of mRNA expression and constitute an important fraction of the human transcriptome. Furthermore, there is a growing number of studies that have reported that lncRNAs play important roles in tumor genesis, progression, and metastasis, as well as many other cellular processes (13–16). Aberrant expression of lncRNAs may contribute to glioma pathogenesis, including cellular proliferation, apoptosis, and metastasis (17–20). The dysregulation of lncRNAs may serve as diagnostic biomarkers of early stages of glioma and could be exploited as therapeutic targets (21–23). However, the potential pathological and biological roles of lncRNAs and mRNAs in different degrees of glioma malignancy have yet to be elucidated.

Recently, the deep sequencing of transcriptomes is being utilized with a higher sensitivity for the identification of differential expression. Advances in next-generation, deep-sequencing technology have identified a number of ncRNAs. Here, we performed high-throughput transcriptome sequencing of glioma tissues and normal tissues to determine lncRNA and mRNA profiles, to investigate novel tumor-related lncRNAs and mRNAs in glioma, and to generate model profiles for future studies. We constructed a gene co-expression network for the differentially expressed lncRNAs and mRNAs in glioma tissues to further investigate the relationship between lncRNAs and mRNAs. We also conducted gene-ontology (GO) enrichment analysis and pathway-enrichment analysis for the differentially expressed lncRNAs and mRNAs. In addition, the five most differentially expressed lncRNAs and mRNAs were verified by quantitative real-time PCR (qRT-PCR).

## Materials and Methods

### Patients and Samples

We recruited 50 patients diagnosed with glioma or epilepsy and collected their tumor or normal tissues from March 2014 to December 2018. All the tumor patients were explicitly diagnosed with glioma by histopathological examination after surgery and were classified as grade II, grade III, and grade IV according to the CNS tumor-classification criteria (fourth edition) published by the WHO in 2016. Six normal tissues and 16 glioma tissues (grade II, six cases; grade III, four cases; and grade IV, six cases) were selected at random for high-throughput transcriptome sequencing, and qRT-PCR analysis was performed in the other samples. All the patients had no prior chemotherapy or radiotherapy and did not have any other serious diseases. The brain tissues for RNA-sequencing were transferred to −80°C storage within 60 min of resection. This experiment was approved by the Ethics committee of Nantong University, and all patients provided informed consent.

### RNA Library Construction and RNA Sequencing

Total RNA was extracted from the brain tissue samples using Trizol reagent (Invitrogen, Cat no.15596-026, USA), following the manufacturer's protocol. Ribosomal RNA was removed from the total RNA samples using Ribo-Zero rRNA Removal Kits (Illumina, USA), as per the manufacturer's instructions. RNA libraries were constructed using rRNA-depleted RNAs with TruSeq Stranded Total RNA Library Prep Kit (Illumina, USA), according to the manufacturer's instructions. The libraries were controlled for quality and quantified using the BioAnalyzer 2,100 system (Agilent Technologies, USA). Then, the 10-pM libraries were denatured as single-stranded DNA molecules, captured on Illumina flow cells, amplified *in situ* as clusters, and finally amplified (150 cycles) and sequenced on an Illumina HiSeq Sequencer, according to the manufacturer's instructions.

The high-quality reads generated were aligned to the human reference genome (UCSC hg19) with hisat2 software. Then, guided by the Ensembl gene-annotation file, cuffdiff software (part of cufflinks) was used to reveal the expression profile of the lncRNAs and mRNAs in terms of Fragments Per Kilobase of transcript per Million mapped reads (FPKM) values, from which the fold change between groups and the corresponding *p*-values were calculated. Subsequently, differentially expressed lncRNAs and mRNAs were identified and lncRNA target genes were predicted by their locations to nearby genes.

### Series Test of Clusters (STC)

STC analysis was used to screen the significant trending models associated with glioma and their corresponding differentially expressed mRNAs and lncRNAs. Fisher's exact test was used to identify significant profiles, and *p* ≤ 0.05 was used as a threshold of significance.

### Gene Co-expression Analyses

To explore the interactions between the DEGs and differentially expressed lncRNAs, gene co-expression networks were build based on their co-expression patterns. The lncRNAs with a related coefficient of *R* ≥ 0.95 or *R* ≤ −0.95 were screened for functional analysis.

### Gene Ontology (GO) Analysis

All differentially expressed genes (DEGs) were mapped to GO terms in the GO database (http://www.geneontology.org/). A hypergeometric test was applied to find significantly enriched GO terms in the input list of DEGs, based on “GO::TermFinder” (http://smd.stanford.edu/help/GO-TermFinder/GO_TermFinder_help.shtml).

A Bonferroni correction was applied to adjust the *p*-value. The false discovery rate (FDR)-adjusted *p* ≤ 0.05 was used as a threshold and GO terms fulfilling this condition were defined as significantly enriched.

### Pathway Analysis

Pathway analysis was used to identify pathways involving the DEGs, according to the Kyoto Encyclopedia of Genes and Genomics (KEGG). Pathways with FDR-adjusted *p* ≤ 0.05 were defined as significantly enriched. Cytoscape was used to generate graphical representations of the pathways.

### Quantitative Real-Time Polymerase Chain Reaction (qRT-PCR)

Reverse transcription was performed with the High-Capacity cDNA Reverse Transcription Kits (Applied Biosystems, Foster City, USA), according to the manufacturer's instructions. qRT-PCR was performed on an ABI 7500 thermocycler (Applied Biosystems, Foster City, CA, USA) by using SYBR Green Real-Time PCR Master Mix (Toyobo, Japan). GAPDH was used for normalization. All qPCR reactions were performed in biological triplicates. Primer sequences are listed in Supplemental Table 1.

**Table 1:** The 50 most significantly differentially expressed lncRNAs.

**Transcript_ID**	**Gene_ID**	**Log_**2**_ (fold change)**	***p*-value**	**Regulation**
TCONS_l2_00004574	XLOC_l2_002352	25.4352	0.00645	Up
uc031tga.1	BC018860	9.58718	0.01625	Up
ENST00000412788	ENSG00000130600	6.97233	0.02285	Up
ENST00000608521	ENSG00000227195	6.40785	0.02125	Up
uc022adp.1	GU228584	5.95802	0.0006	Up
ENST00000601079	ENSG00000227195	5.85652	0.0208	Up
ENST00000549278	ENSG00000257156	−5.71737	0.0278	Down
ENST00000510667	ENSG00000249307	5.59395	0.028	Up
ENST00000518934	ENSG00000254139	5.3137	0.00855	Up
ENST00000451368	ENSG00000225792	5.30241	0.0039	Up
ENST00000597267	ENSG00000227195	5.21331	0.01125	Up
uc011ktp.2	FAM115C	5.00654	0.0466	Up
ENST00000511634	ENSG00000248184	5.00535	0.03295	Up
ENST00000509088	ENSG00000249307	4.92487	0.0293	Up
NR_024443	LOC100133920	4.89535	0.0483	Up
ENST00000413670	ENSG00000225206	−4.88674	0.02905	Down
ENST00000438049	ENSG00000231419	4.81286	0.00125	Up
ENST00000608254	ENSG00000233067	4.81252	0.0275	Up
ENST00000537762	ENSG00000256542	−4.74613	0.01925	Down
NR_034142	LHFPL3-AS1	4.56925	0.01805	Up
ENST00000363359	ENSG00000265185	4.46038	0.0187	Up
ENST00000413238	ENSG00000231419	4.43472	0.0242	Up
TCONS_00029193	XLOC_013852	4.36865	0.00005	Up
uc021uec.1	EPR-1	4.26132	0.03495	Up
ENST00000447563	ENSG00000231419	4.25414	0.00375	Up
ENST00000593438	ENSG00000253552	4.23103	0.0426	Up
ENST00000590421	ENSG00000267280	4.21325	0.0351	Up
ENST00000555772	ENSG00000258754	4.18705	0.02865	Up
ENST00000427722	ENSG00000235326	4.13483	0.0311	Up
ENST00000423456	ENSG00000214548	−4.13843	0.0123	Down
ENST00000518865	ENSG00000248690	4.11723	0.0477	Up
ENST00000422842	ENSG00000234173	4.10453	0.002	Up
ENST00000442411	ENSG00000224057	4.07224	0.0022	Up
ENST00000581282	ENSG00000266045	−4.0662	0.0117	Down
uc003tqn.3	EGFR	4.05537	0.00225	Up
ENST00000262952	ENSG00000034063	4.04982	0.03855	Up
ENST00000432171	ENSG00000226786	4.0389	0.03535	Up
ENST00000438810	ENSG00000224271	−4.03373	0.04935	Down
ENST00000603284	ENSG00000270866	4.02231	0.01445	Up
ENST00000522718	ENSG00000253161	−3.94716	0.028	Down
TCONS_00006930	XLOC_002759	3.93013	0.04995	Up
ENST00000425277	ENSG00000228133	−3.91056	0.00215	Down
uc001zdv.3	DQ786262	3.8969	0.0171	Up
ENST00000520255	ENSG00000254235	3.89324	0.03705	Up
TCONS_00005474	XLOC_003316	3.86985	0.01935	Up
ENST00000563044	ENSG00000260978	3.85627	0.02885	Up
NR_015364	LOC441204	3.85363	0.0083	Up
ENST00000423403	ENSG00000231252	3.75703	0.01055	Up
ENST00000534856	ENSG00000255931	−3.73183	0.0193	Down
ENST00000499452	ENSG00000245954	−3.71881	0.0161	Down

*Transcript_ID, the transcript ID; Gene_ID, the Ensembl gene identifier; Log_2_ (fold change), fold change between the two groups; p_value, p-value between the two groups; regulation, “up” indicates upregulation, and “down” indicates downregulation*.

### Statistical Analysis

Student's *t*-tests and one-way analyses of variance (ANOVA) with Bonferroni corrections for multiple comparisons were performed to determine significant differences between different groups. A false discovery rate (FDR)-adjusted *p* ≤ 0.05 was regarded as statistically significant. All statistical details are specified in the figure legends.

## Results

### Differentially Expressed lncRNAs and mRNAs in Glioma Tissues Compared With Normal Tissues

**Figure 1:**
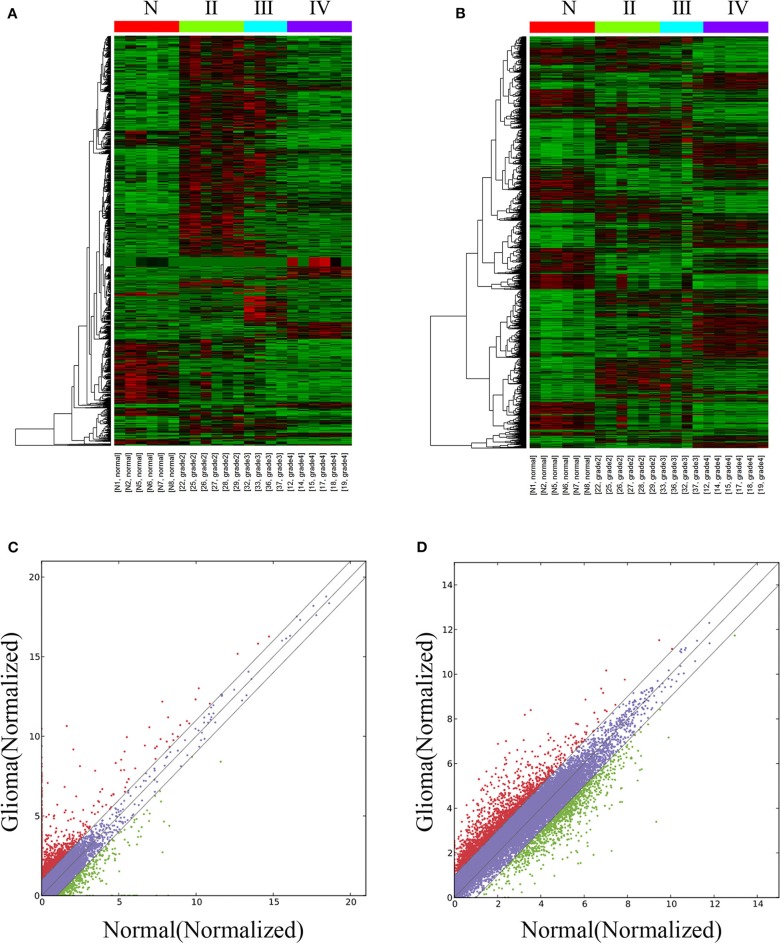
Differentially expressed lncRNAs and mRNAs in glioma tissues and normal tissues. The hierarchical clustering and heat map show differential lncRNA **(A)** and mRNA **(B)** expression profiles of all targets among samples: red represents high relative expression, and green represents low relative expression. The variation of differential lncRNAs and mRNAs between the glioma and normal groups is shown in a scatter plot **(C,D)**. The red dots and green dots denote a fold change >2.

The lncRNA and mRNA expression levels were compared in glioma tissues and normal tissues. We found 578 lncRNAs and 3,216 mRNAs that were significantly dysregulated in glioma tissues. Among these, 509 lncRNAs and 2,282 mRNAs were upregulated and 69 lncRNAs and 934 mRNAs were downregulated (fold change ≥ 2.0, *p* < 0.05). We then used hierarchical-clustering analysis to reveal between-group comparisons of lncRNA and mRNA expression levels (Figures 1A,B). In addition, the variation of differential lncRNAs and mRNAs between the glioma and normal groups is shown in a scatter plot (Figures 1C,D). The 30 most significantly differentially expressed lncRNAs and mRNAs are listed in Tables 1, 2. The 20 most significantly differentially expressed lncRNAs and mRNAs in Grade II, III, and IV are listed in Tables 3, 4.

**Table 2:** The 50 most significantly differentially expressed mRNAs.

**Gene**	**Gene_ID**	**Log_**2**_ (fold change)**	***p*-value**	**Regulation**
NPTX2	ENSG00000106236	−6.07976	0.00005	Down
HIST2H3C	ENSG00000203811	5.63959	0.012	Up
LAMB3	ENSG00000196878	−5.55463	0.00005	Down
MMP10	ENSG00000166670	−5.42366	0.00355	Down
HIST1H1B	ENSG00000184357	5.25975	0.0024	Up
GSTM1	ENSG00000134184	5.19776	0.00005	Up
ADAMDEC1	ENSG00000134028	5.12898	0.00005	Up
TOP2A	ENSG00000131747	5.10747	0.00005	Up
KLRC2	ENSG00000205809	5.10461	0.00065	Up
BCAN	ENSG00000132692	5.07955	0.00005	Up
DLL3	ENSG00000090932	5.00055	0.00005	Up
EGFR	ENSG00000146648	4.99159	0.00005	Up
HS3ST2	ENSG00000122254	−4.95242	0.0121	Down
HIST1H3B	ENSG00000124693	4.91151	0.0029	Up
ACRC	ENSG00000147174	−4.87204	0.00005	Down
BTBD17	ENSG00000204347	4.80482	0.00435	Up
HIST1H2BO	ENSG00000196331	4.7694	0.00105	Up
KLRC4	ENSG00000183542	4.62022	0.0231	Up
AL450307.1	ENSG00000189275	−4.61135	0.007	Down
IRX2	ENSG00000170561	4.54101	0.00015	Up
NEU4	ENSG00000204099	4.53795	0.00005	Up
F5	ENSG00000198734	4.53494	0.00005	Up
NTS	ENSG00000133636	4.52864	0.0084	Up
NEK2	ENSG00000117650	4.47922	0.0003	Up
TNFRSF6B	ENSG00000243509	−4.47257	0.0445	Down
CENPA	ENSG00000115163	4.46647	0.00015	Up
CHST9	ENSG00000154080	4.31116	0.0002	Up
COL20A1	ENSG00000101203	4.29479	0.00005	Up
NKAIN4	ENSG00000101198	4.22354	0.00005	Up
RPE65	ENSG00000116745	4.22305	0.00345	Up
HIST1H3C	ENSG00000196532	4.21731	0.0029	Up
SLC38A4	ENSG00000139209	4.20996	0.00105	Up
PBK	ENSG00000168078	4.18686	0.0012	Up
NDC80	ENSG00000080986	4.17834	0.0016	Up
AQP1	ENSG00000240583	4.17639	0.00025	Up
PDYN	ENSG00000101327	−4.16504	0.00165	Down
PCDH15	ENSG00000150275	4.15302	0.00005	Up
POSTN	ENSG00000133110	4.12155	0.00005	Up
FAM27E1	ENSG00000237198	4.11957	0.03865	Up
OR2B2	ENSG00000168131	4.0847	0.00965	Up
HIST1H2AM	ENSG00000233224	4.05657	0.00415	Up
GNAT1	ENSG00000114349	4.04505	0.00065	Up
SMOC1	ENSG00000198732	4.03992	0.00005	Up
ADCYAP1	ENSG00000141433	−4.03415	0.00005	Down
HAPLN1	ENSG00000145681	4.01781	0.00005	Up
HIST2H4A	ENSG00000183941	4.00519	0.0003	Up
IGFBP2	ENSG00000115457	3.99938	0.00015	Up
MYBL2	ENSG00000101057	3.99309	0.00795	Up
CDCA2	ENSG00000184661	3.97406	0.0001	Up
PRLHR	ENSG00000119973	3.97111	0.00155	Up

*Gene, the gene symbol; Gene_ID, the Ensembl gene identifier; Log_2_ (fold change), fold change between the two groups; p_value, p-value between the two groups; regulation, “up” indicates upregulation, and “down” indicates downregulation*.

**Table 3:** The 20 most significantly differentially expressed lncRNAs in Grade II, III, and IV.

**Grade II**		**Grade III**		**Grade IV**	
**Transcript_ID**	**Log_**2**_ (fold change)**	**Transcript_ID**	**Log_**2**_ (fold change)**	**Transcript_ID**	**Log_**2**_ (fold change)**
TCONS_l2_00004574	24.3613	TCONS_l2_00004574	25.2656	TCONS_l2_00004575	−173.563
ENST00000507761	15.4384	NR_028272	−21.3918	TCONS_l2_00004574	26.1453
ENST00000439232	9.16074	uc031tga.1	10.8534	ENST00000414790	16.2916
ENST00000429008	7.36223	ENST00000601079	7.41343	ENST00000452769	−14.1961
ENST00000518934	6.71654	ENST00000597267	6.75795	ENST00000439232	8.48864
ENST00000509088	6.29573	uc022adp.1	6.31318	TCONS_l2_00004577	−8.28525
ENST00000511634	6.00731	ENST00000426965	6.26577	ENST00000534856	−6.48133
ENST00000451368	5.60166	TCONS_00023458	6.15915	ENST00000427775	−6.12229
ENST00000507491	5.48005	ENST00000453569	6.00558	TCONS_00024611	−5.7225
ENST00000549278	−5.42917	TCONS_00029193	5.89461	ENST00000525363	5.59857
NR_120607	−5.25235	ENST00000496499	5.87566	ENST00000554205	−5.52642
ENST00000413670	−5.08876	ENST00000451368	5.87474	ENST00000568267	−5.4216
ENST00000534856	−4.95754	ENST00000450618	5.84935	ENST00000511849	−5.35894
ENST00000519821	4.93422	ENST00000496478	5.83764	ENST00000581282	−5.22225
ENST00000534584	−4.91577	ENST00000413238	5.6796	ENST00000555928	−5.21035
ENST00000426585	4.90462	ENST00000475999	5.59518	ENST00000499452	−5.17663
ENST00000535911	4.84049	ENST00000547748	5.59129	ENST00000509844	−5.16697
ENST00000499452	−4.82772	TCONS_00029735	5.51277	ENST00000262952	5.08551
NR_105016	4.52243	ENST00000579362	5.4347	uc021yib.1	−5.07134
ENST00000432171	4.48445	ENST00000447563	5.432	TCONS_00024610	−5.07118

*Transcript_ID, the transcript ID; Log_2_ (fold change), fold change between the two groups*.

**Table 4:** The 20 most significantly differentially expressed mRNAs in Grade II, III, and IV.

**Grade II**		**Grade III**		**Grade IV**	
**Gene**	**Log_**2**_ (fold change)**	**Gene**	**Log_**2**_ (fold change)**	**Gene**	**Log_**2**_ (fold change)**
AC003006.7	33.3807	C8B	8.21525	AC003006.7	34.1215
NKG2-E	14.6013	ADAMDEC1	6.14045	CARTPT	−6.44813
MMP3	−8.53564	LAMB3	−6.08015	PVALB	−6.30211
TNFRSF6B	−8.17738	GSTM1	5.80422	AL450307.1	−6.28653
NPTX2	−6.92934	NPTX2	−5.76227	SERTM1	−6.25538
KLRC2	6.28025	MMP10	−5.75584	HIST1H1B	6.21729
SFN	−6.19581	DLL3	5.6937	FAM153C	−6.04122
CHI3L1	−6.00804	NEU4	5.6856	NPAS4	−6.0054
F5	5.96362	BCAN	5.62658	HIST2H3C	5.99044
MMP10	−5.92447	GNAT1	5.51854	TOP2A	5.96605
TIMP1	−5.72832	COL20A1	5.44559	FAM153A	−5.8515
LAMB3	−5.66232	HOXA10	5.30305	HIST1H3B	5.82345
KLRC4	5.57167	DAPL1	5.29865	NPTX2	−5.81806
PRLHR	5.41416	ACRC	−5.23327	EGFR	5.81172
ADCYAP1	−5.36875	CDK6	5.23096	HIST1H2BO	5.70465
AL450307.1	−5.36673	AC005544.1	5.1904	HIST1H4L	5.64717
PTGS2	−5.30548	OOSP2	5.15603	POSTN	5.63341
NPAS4	−5.25036	EGFR	5.12739	TCERG1L	−5.60801
IRX2	5.19441	CTD-2021H9.3	5.09933	WIF1	−5.44568
SERTM1	−5.15551	C19orf80	5.08284	NRGN	−5.41262

*Gene, the gene symbol; Log_2_ (fold change), fold change between the two groups*.

### Model Profile Analysis of lncRNAs and mRNAs in Glioma Tissues and Normal Tissues

**Figure 2:**
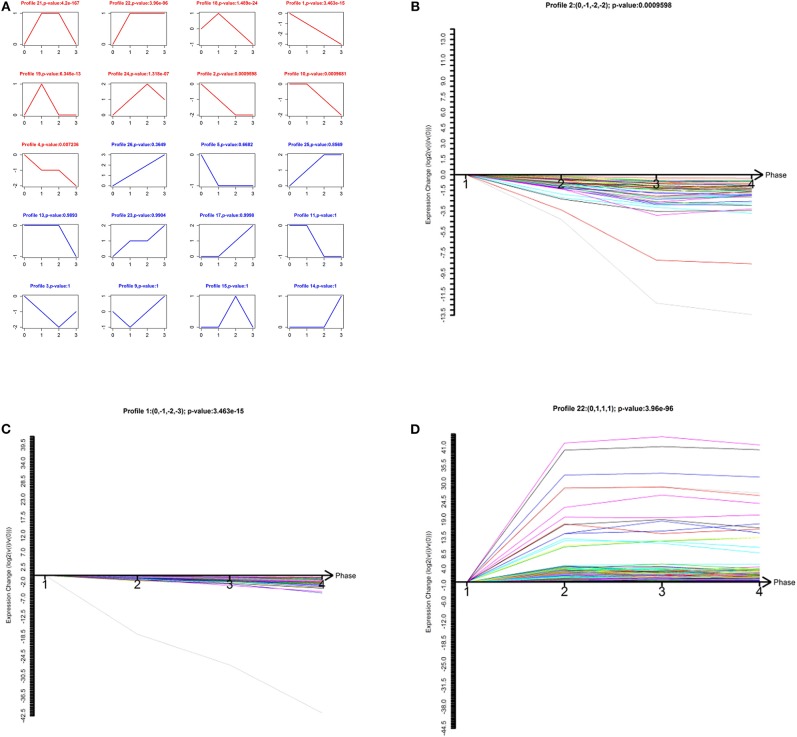
Model-profile analysis for differentially expressed lncRNAs associated with the malignancy-grades of gliomas. **(A)** The expression patterns of differentially expressed lncRNAs were analyzed and 20 model profiles were summarized. Each model-expression profile is represented by a box. In total, nine expression patterns of lncRNAs exhibited significant *p*-values (*p* < 0.05) and the red boxes denote the changing trend of these significant profiles. **(B–D)** The patterns of profiles that have clinical significance are presented.

To narrow down the number of highly significant differentially expressed lncRNAs and mRNAs, we further analyzed their specific expression patterns. Twenty model profiles of lncRNAs and 10 model profiles of mRNAs were summarized. Among the 20 patterns, we identified nine patterns of lncRNAs that exhibited significant *p*-values (Figure 2A; *p*-values in red boxes).

Among the nine significant patterns, the expression of lncRNAs in profile No. 1, 2, and 22 were of clinical significance (Figures 2B–D). The lncRNA model profile No.1 and No. 2 contained 78 lncRNAs and 58 lncRNAs, respectively, the expressions of which were decreased consistently in glioma tissues (grades II–IV). Additionally, lncRNA profile No. 22 was constructed with 457 lncRNAs, which exhibited consistently up-regulated expression in glioma tissues (grades II–IV).

### Establishment of the Gene Co-expression Network for lncRNAs and mRNAs in Glioma Tissues

**Figure 3:**
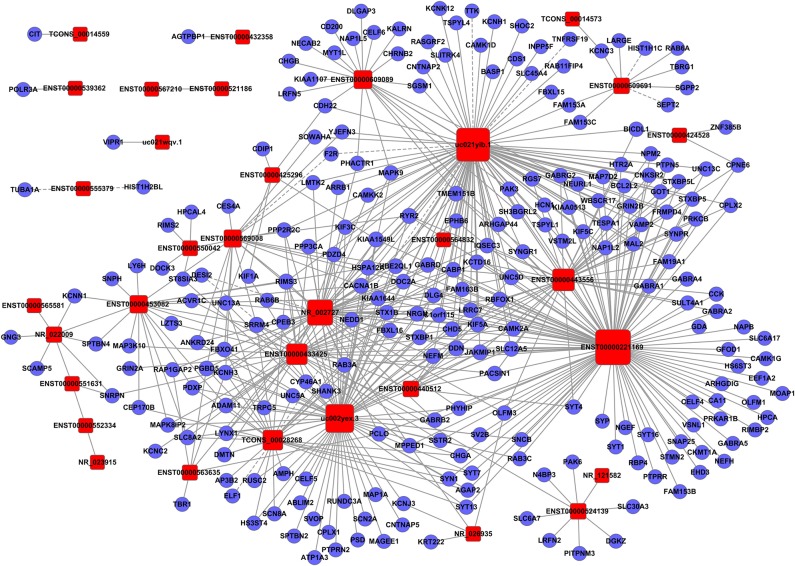
Establishment of the gene co-expression network for lncRNAs in profile No. 1 and the corresponding mRNAs. Here, 33 square nodes and 244 circular nodes represent lncRNAs and mRNAs, respectively. The edges show the interaction between the lncRNAs and mRNAs.

**Figure 4:**
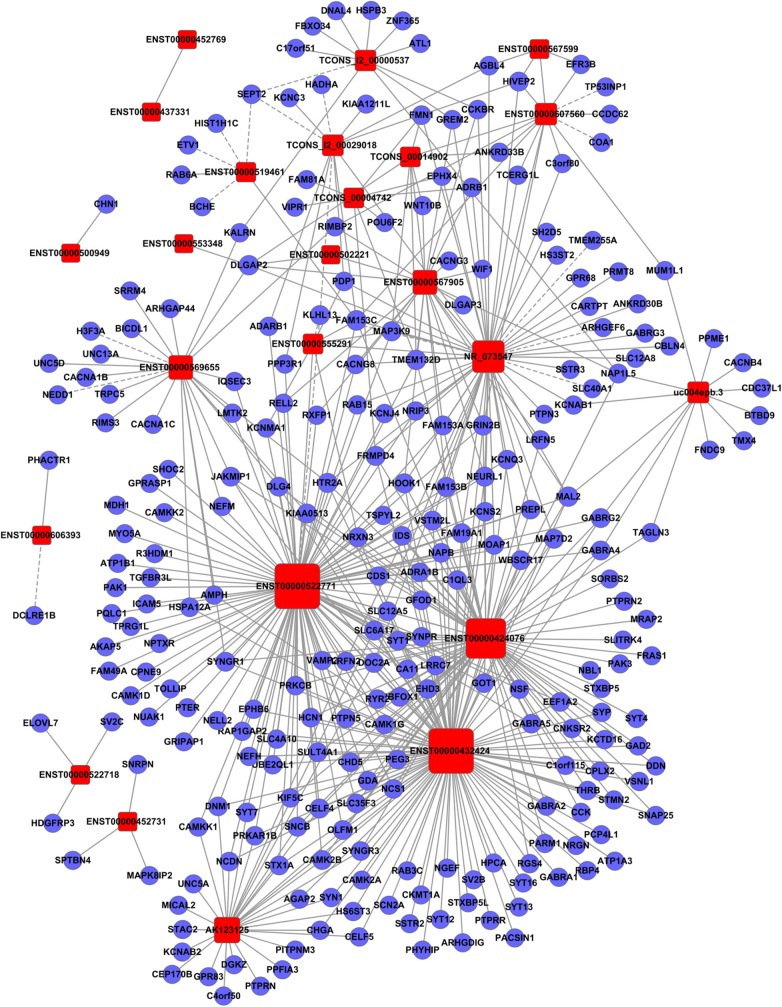
Establishment of the gene co-expression network for lncRNAs in profile No. 2 and the corresponding mRNAs. Here, 24 square nodes and 258 circular nodes represent lncRNAs and mRNAs, respectively. The edges show the interaction between the lncRNAs and mRNAs.

**Figure 5:**
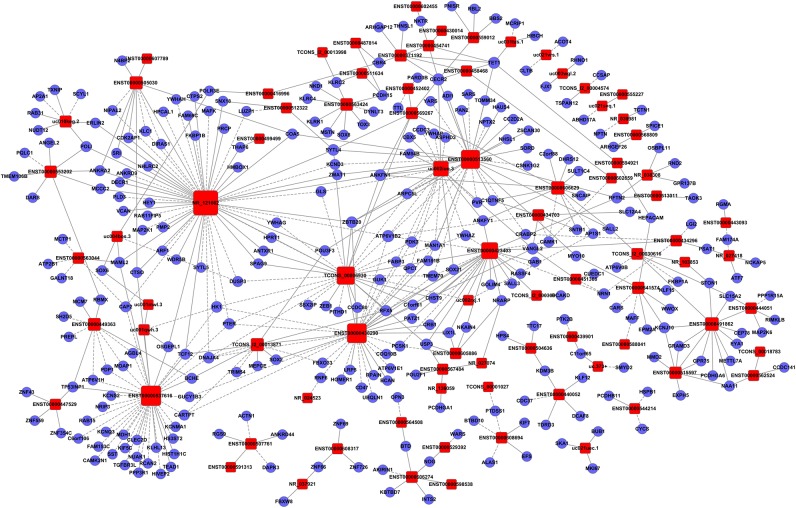
Establishment of the gene co-expression network for lncRNAs in profile No. 22 and the corresponding mRNAs. Here, 83 square nodes and 287 circular nodes represent lncRNAs and mRNAs, respectively. The edges show the interaction between the lncRNAs and mRNAs.

To clarify the relationship between lncRNAs and mRNAs in glioma, we performed correlation analyses for lncRNAs and mRNAs in terms of their expression values in glioma tissues. Additionally, a gene co-expression network between mRNAs and lncRNAs was constructed (Figures 3–5). In profile No. 22, there were 83 square nodes and 287 circular nodes that represented lncRNAs and mRNAs, respectively. Moreover, the edges showed the interaction between the lncRNAs and mRNAs. These results indicated that lncRNAs may play vital roles in the pathogenesis of glioma.

### GO- and Pathway-Enrichment Analyses of Differentially Expressed lncRNAs

**Figure 6:**
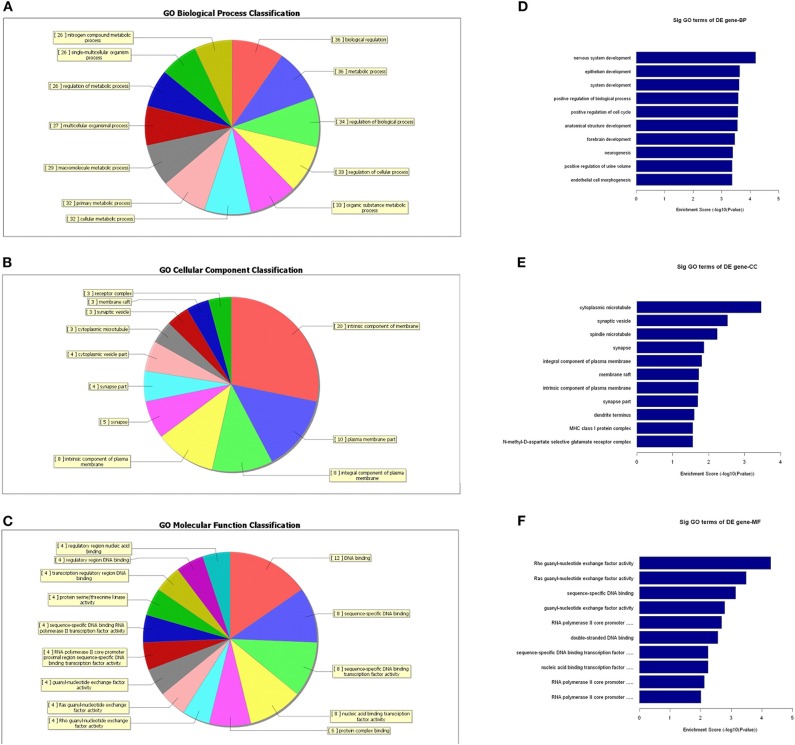
GO-enrichment analysis of differentially expressed lncRNAs. Each category (BP, CC, and MF) has its classifications and the lncRNAs associated with each category were counted and visualized as pie charts **(A–C)**. The 10 most enriched GO terms (in descending order of enrichment score) in the three categories are shown **(D–F)**.

To further identify the functional roles of these differential lncRNAs found to be dysregulated in the tumor group, we conducted GO-enrichment and pathway-enrichment analyses. The GO analysis returned terms associated with three categories: molecular function (MF), cellular component (CC), and biological process (BP). The number of lncRNAs in profile No. 22 found associated with each GO term was counted and are shown in a pie chart (Figure 6). The 10 most enriched GO terms (in descending order of enrichment score) within the three categories are shown in Figures 6D–F.

**Figure 7:**
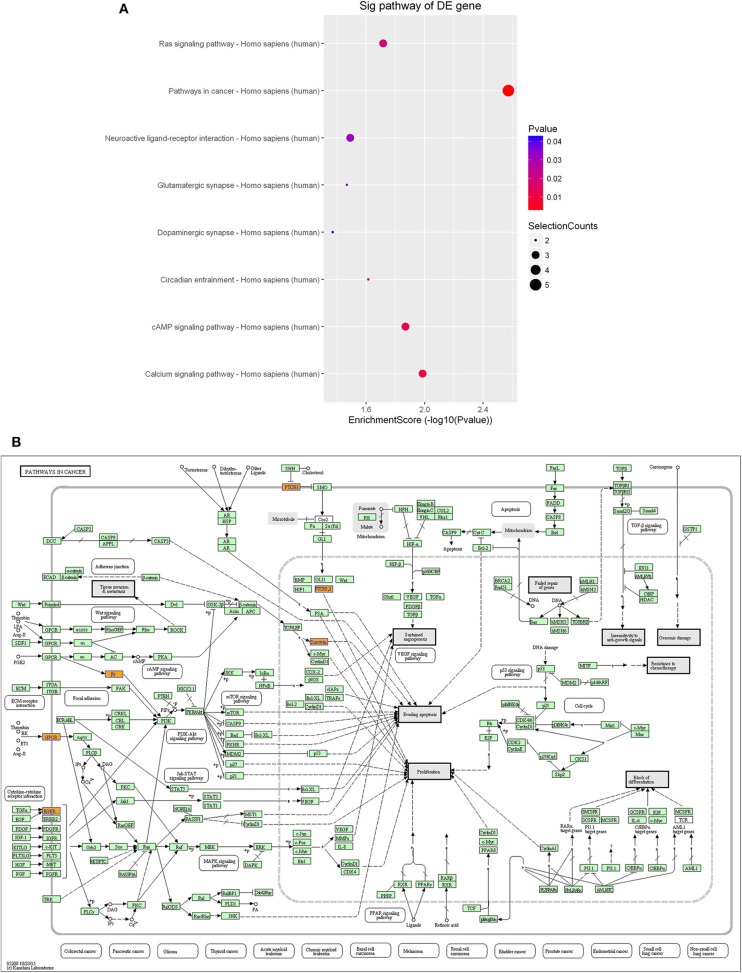
Pathway-enrichment analysis of differentially expressed lncRNAs. The dot plot shows the top-eight enrichment scores (–log_10_ *p*-value) of the significantly enriched pathways **(A)** and the regulatory roles of the lncRNAs involved in the pathways in cancer. **(B)** Nodes in orange are associated with up-regulated enriched genes, whereas green nodes denote genes that showed no statistical significance.

Furthermore, KEGG pathway analysis demonstrated that the differentially expressed lncRNAs were significantly enriched in various important pathways. The dot plot in Figure 7A shows the eight highest enrichment scores (lowest log_10_ *p*-values) of the significant pathways (Figure 7A), and Figure 7B shows the regulatory roles of the lncRNAs involved in cancer pathways.

### GO- and Pathway-Enrichment Analyses of Differentially Expressed mRNAs

**Figure 8:**
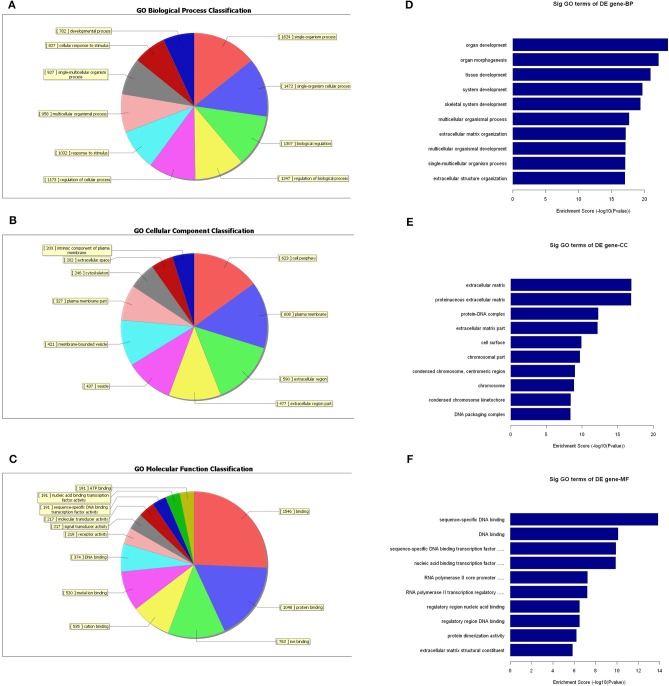
GO-enrichment analysis of differentially expressed mRNAs. Each GO category has many different related terms and the mRNAs associated with each term were counted and visualized as pie charts. **(A–C)** The 10 most enriched GO terms (in descending order of enrichment score) of the three categories are shown **(D–F)**.

Since the functions of the differentially expressed mRNAs are different from those of the lncRNAs, we conducted independent GO-enrichment and pathway-enrichment analyses for the mRNAs. Figure 8 shows the number of mRNAs associated with each GO term (Figures 8A–C), and the 10 most enriched GO terms (in descending order of enrichment score) in each of the three categories are shown in Figures 8D–F.

**Figure 9:**
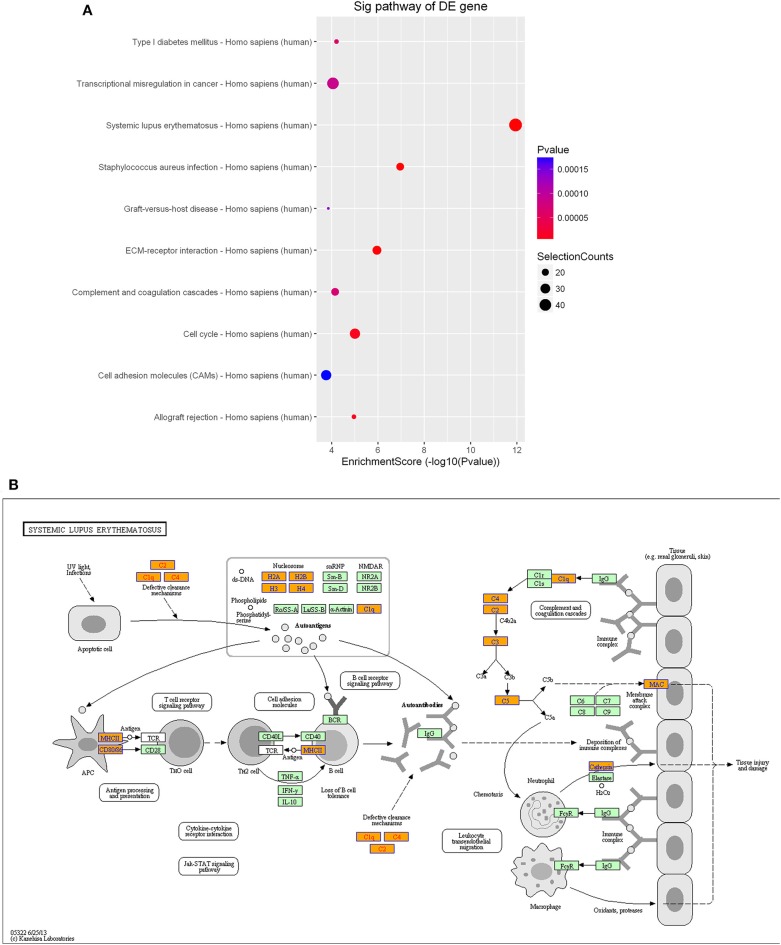
Pathway-enrichment analysis of differentially expressed mRNAs. The dot plot shows the top-ten enrichment scores (–log_10_ *p*-values) of the significantly enriched pathways **(A)** and the regulatory roles of the mRNAs involved in systemic lupus erythematosus. **(B)** Orange nodes are associated with up-regulated enriched genes, whereas green nodes denote genes that showed no statistical significance.

As observed with the lncRNAs, KEGG pathway analysis demonstrated that the differentially expressed mRNAs were significantly enriched in various important pathways. The dot plot shows the eight most significantly enriched pathways (enrichment score = –log_10_ *p*-value) (Figure 9A), and the regulatory roles of the mRNAs involved in systemic lupus erythematosus and the staphylococcus aureus infections are shown in Figure 9B.

### Validation of Sequencing Results by qRT-PCR

**Figure 10:**
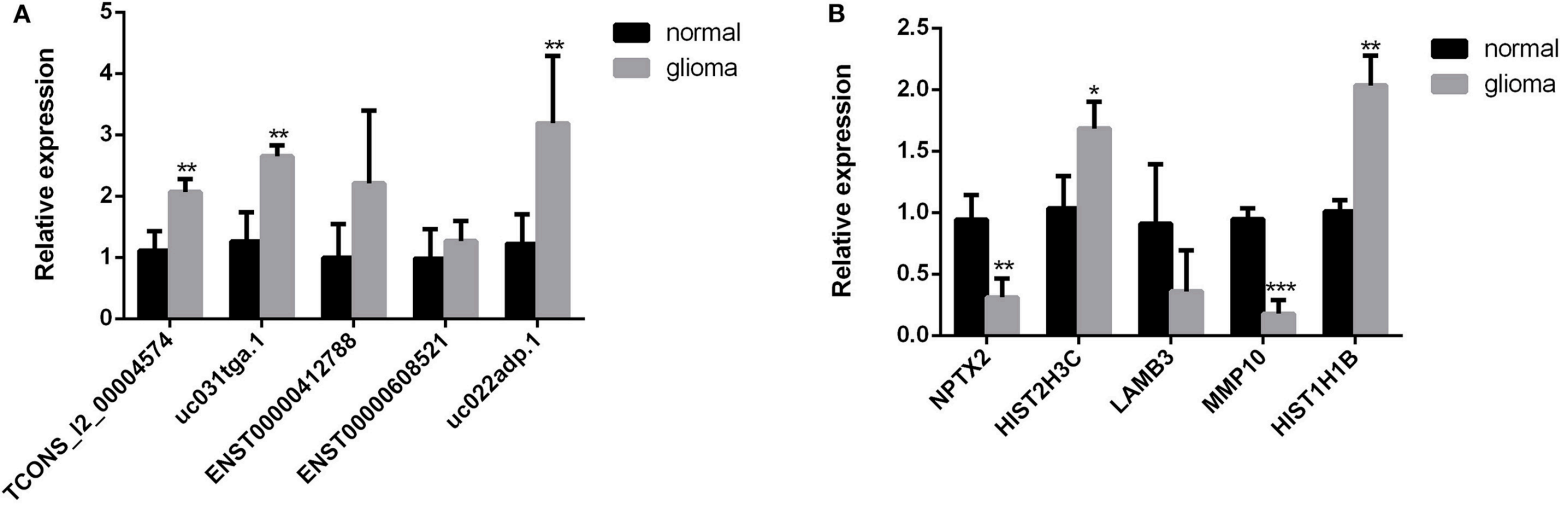
Validation of sequencing results by qRT-PCR. Three of the five lncRNAs (TCONS_l2_00004574, uc031tga.1, and uc022adp.1) **(A)** and four of the five mRNAs (NPTX2, HIST2H3C, MMP10, and HIST1H1B) **(B)** were found to be differentially expressed in the glioma samples, which were in agreement with our sequencing results. ^*^P < 0.05, ^**^P < 0.01, ^***^P < 0.001.

To validate the sequencing data and bioinformatic results, the five most differentially expressed lncRNAs (TCONS_l2_00004574, uc031tga.1, ENST00000412788, ENST00000608521, and uc022adp.1) and mRNAs (NPTX2, HIST2H3C, LAMB3, MMP10, and HIST1H1B) were selected for qRT-PCR. In agreement with our sequencing results, three of the five lncRNAs (TCONS_l2_00004574, uc031tga.1, and uc022adp.1) and four of the five mRNAs (NPTX2, HIST2H3C, MMP10, and HIST1H1B) were found to be differentially expressed in the glioma samples (*P* < 0.05; Figure 10).

## Discussion

As new research techniques have developed, a growing number of high-throughput platforms have been used to analyze gene expression in glioma. However, previous studies mostly focused on mRNA, DNA, or protein levels in glioblastoma or high-grade glioma using proteomics or microarrays (24–26). In the present study, we assessed the significantly differentially expressed lncRNAs and mRNAs between normal tissues and glioma tissues using high-throughput sequencing. To our knowledge, this study is the first to identify the pathological grade-associated transcriptome profiles of lncRNAs and mRNAs in glioma. We identified 578 lncRNAs and 3,216 mRNAs that were significantly dysregulated in glioma tissues. Among these, 509 lncRNAs and 2,282 mRNAs were upregulated, whereas 69 lncRNAs and 934 mRNAs were downregulated (fold change ≥ 2, *p* < 0.05). This result indicated that these lncRNAs and mRNAs may be involved in glioma initiation and/or progression. However, further research is required to elucidate the detailed mechanisms of the involvement of these lncRNAs and mRNAs in glioma.

The lncRNAs and mRNAs that dynamically changed with differing degrees of glioma malignancy may play crucial biological roles in the disease process (27, 28). Through model-profile analysis, the dynamic expression profiles of lncRNAs and mRNAs were obtained, and the nine significant dynamic expression profiles of lncRNAs were then screened. The three profiles of clinical significance were profiles No. 1, No. 2, and No. 22. Each of these profiles contained a large number of lncRNAs that were consistently down- or up-regulated in the different grades of glioma tissues. In the most significant profile (No. 22), seven lncRNAs were identified to be involved in viral carcinogenesis. The lncRNAs with significant dynamic-expression changes may be more correlated with the malignancy of glioma and, hence, may play vital roles in the regulation of glioma initiation and progression.

To further discern the key lncRNAs associated with glioma, we integrated lncRNA and mRNA co-expression networks in profiles No. 1, No. 2, and No. 22. In total, 83 lncRNAs and 287 mRNAs were identified to play vital regulatory roles in model profile No. 22. Many studies have reported that a large number of lncRNAs, such as ZEB1-AS1 and PCNA-AS1 (29, 30) played important roles in the accurate and complicated co-expression networks. We screened a number of lncRNAs and mRNAs that included key genes that are closely associated with the pathogenesis of glioma. For example, SOX21 has been reported to be closely related to the tumorigenesis of glioblastoma, hepatocellular carcinoma, and colorectal cancer (31–33). This suggests that these co-expressed lncRNAs and mRNAs may participate in cancer-related pathways. It was also found that most lncRNAs can be co-expressed with various mRNAs, indicating that each lncRNA may regulate many mRNAs.

A growing number of lncRNAs have been identified in tumors (34, 35); however, the various functions of lncRNAs still remain poorly characterized. To infer the functional roles of the lncRNAs in glioma, GO and pathway analyses were performed for these differentially expressed lncRNAs. The GO analysis indicated that these lncRNAs are most significantly enriched in domains of “biological regulation,” “metabolic process,” and “regulation of biological process,” which are strongly associated with glioma. In addition, the pathway analysis revealed that “pathway in cancer,” “calcium signaling pathway,” “cAMP signaling pathway,” and “Ras signaling pathway” are related with the pathogenic process of glioma.

In addition, there were also some limitations of our present study. Regardless of the technology performed to detect expression levels and the sample sizes that are used, the actual gene expression levels vary among individuals because expression is a random process. Consequently, the analysis data may not be powerful enough to reflect these actual expression levels across individuals. However, biological variability will decrease with the increase of the scale of samples. Therefore, we will perform further studies with more samples in the future.

## Conclusions

In summary, we characterized the expression profiles of lncRNAs and mRNAs in normal tissues and glioma tissues (WHO grades II–IV). Then, we analyzed the dynamic differentially expressed profiles of lncRNAs and mRNAs, which indicated their potential vital roles in gliomas with different degrees of malignancy. A series of bioinformatics analyses indicated that most of these lncRNAs and mRNAs are involved in important biological processes and pathways associated with the pathogenesis of glioma. These results provide potential directions and valuable resources for future studies via the comprehensive integration of these lncRNAs and mRNAs.

## Data Availability Statement

The datasets generated for this study can be found in GenBank (SRA accession: PRJNA604108).

## Ethics Statement

The studies involving human participants were reviewed and approved by the Ethics committee of Nantong University. The patients/participants provided their written informed consent to participate in this study.

## Author Contributions

All authors listed have made a substantial, direct and intellectual contribution to the work, and approved it for publication.

### Conflict of Interest

The authors declare that the research was conducted in the absence of any commercial or financial relationships that could be construed as a potential conflict of interest.
